# Serum IgA, IgG, IgM levels, total protein (TP), albumin (ALB) changes in elderly hip fracture patients after surgery and nutritional support

**DOI:** 10.5937/jomb0-55434

**Published:** 2025-07-04

**Authors:** Fangqin Jin, Qin Zhang, Jiangping Weng, Xuting Pan, Qinyan Dong, Yuping Zhao

**Affiliations:** 1 The Second Hospital of Jiaxing, Department of Orthopedics, Jiaxing, Zhejiang, China

**Keywords:** nutritional support, stratified nursing, delirium risk assessment, elderly hip fracture, immune function, postoperative delirium, serum IgA, IgG, IgM, total protein, albumin, nutritivna podrška, slojevita nega, procena rizika od delirijuma, stariji pacijenti sa prelomom kuka, imunološka funkcija, postoperativni delirijum, serumski IgA, IgG, IgM, ukupni proteini, albumin

## Abstract

**Background:**

This study aimed to evaluate the effects of nutritional support combined with stratified nursing under delirium risk assessment on immune function and postoperative delirium in elderly hip fracture patients.

**Methods:**

A total of 112 elderly hip fracture patients were divided into two groups: the study group (SG), which received stratified nursing and enteral nutrition, and the control group (CG), which received routine nursing with a normal diet. Key parameters were evaluated and compared, including serum IgA, IgG, IgM levels, total protein (TP), albumin (ALB), postoperative delirium incidence, nutritional status, recovery time, and overall patient outcomes.

**Results:**

Compared to the CG, the SG demonstrated significant improvements in immune function, with increased IgA, IgG, and IgM levels, as well as higher haemoglobin, albumin, and total protein levels. The SG also experienced shorter hospital stays, earlier postoperative mobility, reduced postoperative delirium rates (1.79% vs. 14.29%), lower anxiety and depression scores, better sleep quality, and lower pain scores. Additionally, nursing satisfaction was significantly higher in the SG.

**Conclusions:**

Nutritional support combined with stratified nursing under delirium risk assessment effectively enhances immune function, reduces postoperative delirium, and accelerates recovery in elderly hip fracture patients. This approach may serve as a valuable strategy in postoperative management to improve patient outcomes.

## Abstract (sr)

**Uvod:**

Ova studija imala je za cilj da proceni efekte nutritivne podrške u kombinaciji sa slojevitom negom zasnovanom na proceni rizika od delirijuma na imunološku funkciju i postoperativni delirijum kod starijih pacijenata sa prelomom kuka.

**Metode:**

Ukupno 112 starijih pacijenata sa prelomom kuka podeljeno je u dve grupe: grupu nad kojom je sprovođena studija (SG), koja je primala slojevitu negu i enteralnu ishranu, i kontrolnu grupu (CG), koja je primala rutinsku negu uz standardnu ishranu. Evaluirani su i upoređeni ključni parametri, uključujući nivoe serumskih IgA, IgG i IgM, ukupnih proteina (TP), albumina (ALB), incidenciju postoperativnog delirijuma, nutritivni status, vreme oporavka i ukupne ishode pacijenata.

**Rezultati:**

U poređenju sa CG, SG je pokazala značajna poboljšanja imunološke funkcije, sa povećanim nivoima IgA, IgG i IgM, kao i višim nivoima hemoglobina, albumina i ukupnih proteina. SG je takođe imala kraći boravak u bolnici, raniju postoperativnu mobilnost, nižu stopu postoperativnog delirijuma (1,79% naspram 14,29%), niže skorove anksioznosti i depresije, bolji kvalitet sna i niže skorove bola. Pored toga, zadovoljstvo negom bilo je značajno veće u SG.

**Zaključak:**

Nutritivna podrška u kombinaciji sa slojevitom negom zasnovanom na proceni rizika od delirijuma efikasno poboljšava imunološku funkciju, smanjuje postoperativni delirijum i ubrzava oporavak kod starijih pacijenata sa prelomom kuka. Ovaj pristup može predstavljati značajnu strategiju u postoperativnom menadžmentu za unapređenje ishoda pacijenata.

## Introduction

Hip fracture is a common fracture type in the elderly, and most patients are accompanied by varying degrees of osteoporosis, with a fatality rate of 20% to 40% [1]. With the intensification of population ageing, the occurrence of hip fractures in the elderly is also increasing yearly [2]. Surgery is the main therapy for elderly hip fracture patients, but the risk of postoperative complications is high. Among these, postoperative delirium is one of the common complications in elderly hip fracture patients, and the occurrence of postoperative delirium in elderly hip fracturepatients ranges from 5% to 61% [3]. Postoperative delirium refers to the continuous cognitive disorder after operation, which is clinically manifested as mental disorders such as behavioural changes, memory impairment, and confusion of consciousness [4]. It is a transient encephalopathy syndrome with fluctuating consciousness disorder [5]. Studies have manifested that postoperative delirium in elderly hip fracture patients not only affects physical and mental recovery but also easily induces other complications, including long-term cognitive dysfunction and permanent dementia [6]. Therefore, it is of great significance to prevent and cure postoperative delirium in elderly hip fracture patients.

Delirium risk assessment is an effective method to help medical staff judge the incidence and severity of delirium in patients [7]. The immune system is crucial in postoperative recovery, particularly in elderly patients undergoing hip fracture surgery. Studies have shown that these patients often experience a significant reduction in antimicrobial immune responses post-surgery, making them more susceptible to infections [8]. Immunoglobulins, such as IgA, IgG, and IgM, are essential immune system components and serve as markers of immune response after surgery. Monitoring these immunoglobulin levels can provide insights into a patient’s immune function during recovery.

Serum protein levels, including total protein (TP) and albumin (ALB), are indicators of nutritional status and the body’s ability to recover from surgical stress. Lower admission albumin levels have been independently associated with higher 30-day readmission rates in elderly hip fracture patients, highlighting the importance of maintaining adequate protein levels for optimal recovery [9].

The confusion assessment method of intensive care unit (CAM-ICU), which is commonly used clinically to assess the severity of delirium in critically ill patients, has been proven to be reliable, practical and effective in evaluating the occurrence of postoperative delirium in patients undergoing cardiac surgery, and can effectively decrease the occurrence of delirium in patients and improve prognosis [10]. However, it is rarely used in patients with fractures.

Hip fracture surgery is one of the most traumatic surgical operations [11]. Elderly patients, due to poor body recovery ability and more intraoperative blood loss, are prone to postoperative malnutrition, and nutritional status can affect the occurrence of postoperative complications [12]. Therefore, including nutritional support therapy in the care plan of elderly hip fracture patients not only helps restore functional status, but also promotes patients’ quality of life. Enteral nutrient suspension is a compound preparation, and its main components are water, maltodextrin, whey protein hydrolysate, vegetable oil, vitamins, minerals, and trace elements, along with other essential nutrients for the human body, which is helpful to enhance the postoperative nutritional status of elderly hip fracture patients [13].

This study evaluated the combined impact of nutritional support and stratified nursing under a delirium risk assessment strategy on immune function, postoperative delirium, and overall recovery outcomes in elderly hip fracture patients. By integrating a structured nursing approach based on CAM-ICU scoring with early enteral nutritional intervention, we hypothesised that this strategy would reduce postoperative delirium, improve immune function, enhance nutritional status, and accelerate recovery [14] [15].

## Materials and methods

### General data

One hundred and twelve elderly hip fracture patients surgery who were admitted to our hospital from April 2024 to December 2024 were gathered, followed by dividing into control group (CG) and study group (SG) following the odd and even number of patient admission numbers, with 56 cases in each group.

The hospital’s Medical Ethics Committee approved this study with the patient’s informed consent. Inclusion criteria: (1) Age 65 years; (2) Elderly patients with unilateral hip fracture for the first time, fracture types contained femoral neck fracture, femoral intertrochanteric fracture, along with femoral subtrochanteric fracture; (3) The patient has primary school level or equivalent level or above, and can communicate normally before injury; (4) Mental disorders, cognitive disorders and major organ dysfunction were not diagnosed before surgery. Exclusion criteria: (1) Patients combined with other site fractures; (2) Patients with old hip fracture; (3) Patients with pathological fracture; (4) Patients with acute cardiovascular and cerebrovascular diseases during hospitalisation within six months before admission; (5) Non-surgical treatment of patients; (6) Patients with delirium before surgery; (7) Patients with incomplete medical records or who did not agree to participate in the study.

### Nursing methods

Patients in the CG received routine nursing and were admitted to the hospital for relevant assessment. Before surgery, the patient was given psychological counselling, osteoporosis, hip fracture, preparation matters, precautions for surgery, and prevention of postoperative complications. After surgery, routine health education was provided, such as medication, diet, rehabilitation exercise, and precautions for discharge.

Patients in the SG adopted stratified nursing under the delirium risk assessment strategy based on the CG. First, a nursing team was set up, with a head nurse as the leader and several nursing staff as the team members. The head nurse was responsible for training and managing all team members, including lectures on delirium knowledge, nursing training, content, and using CAM-ICU score sheets, etc. After 1 week of training, qualified nursing staff could be selected through assessment and enter the nursing implementation stage. First of all, the group members established a WeChat group where they could discuss delirium-related knowledge at any time, share the problems encountered in the nursing process, and discuss the best treatment measures in time, which could also help improve the overall professional level of the group members. Secondly, the patients with hip fractures were evaluated and graded using the CAM-ICU rating scale. The risk factors were divided into attention deficit, confusion, repeated fluctuations, acute consciousness changes, and altered consciousness clarity. Low risk: The patient has no risk factors. Medium risk: There are 1 or 2 risk factors. High risk: There are 3 to 4 risk factors. Then, according to the classification, the patient’s bedside was marked accordingly so that the medical staff could know the patient’s relevant situation and give appropriate nursing measures. The head nurse allocated nursing staff to patients of all levels, rationally utilised resources, and maximised the nursing effect. Low-risk patients were equipped with younger nurses, medium-risk patients were equipped with nurses or nurses with rich clinical experience, and high-risk patients were equipped with supervisors or nurses with rich clinical experience. The specific operations were as follows:

(1) Psychological nursing. As the activities of patients with fractures are limited to a certain extent,daily activities need help from others and worry about the prognosis. Hence, the psychological pressure on patients increases, producing various negative emotions and causing delirium. Nursing staff needed to communicate with patients with a soft voice, give psychological guidance and encouragement, explain relevant successful cases and postoperative precautions, complications prevention measures, and establish confidence for patients to treat actively. At the same time, the nursing staff communicated with family members, told them to accompany patients more, and gave spiritual encouragement to eliminate patients’ fear. If the patient’s psychological state was very serious, the nursing staff reported to the doctor and implemented care following the doctor’s advice.

(2) Pain nursing. Patients with hip fractures are often accompanied by severe pain, and if the pain isnot alleviated in time, hallucinations may occur and cause delirium. Therefore, effective pain care should be provided during the nursing process, especially after postoperative anaesthetic withdrawal. The patient’s pain was scored before and after surgery, which could be relieved by shifting attention, changing position, etc. For patients with severe pain, analgesic drugs could be given following the doctor’s advice, and the effects of drugs could be explained at the same time to obtain the patient’s cooperation.

(3) Sleep nursing. If the patient is not well rested, coupled with the fracture site pain and nerve tightness, especially in the elderly, it will cause delirium. Therefore, the nursing staff kept the ward environment comfortable and clean and told patients to go to bed before 21:00 as far as possible. Families could use warm water to wipe patients’ bodies before bed to promote sleep.

(4) Hypoxemia nursing. Hypoxemia can induce brain dysfunction and is closely related to postoperative delirium. Therefore, patients can be given lowflow oxygen after admission to prevent postoperative delirium. Oxygen flow and oxygen inhalation times were adjusted according to patients’ conditions, and vital signs were closely monitored to ensure blood oxygen saturation was above 95%. Atomisation and expectorant drugs could be given to patients with phlegm in the respiratory tract according to the doctor’s advice to ensure blood oxygen saturation of patients.

5) Health education. After the operation, the nursing staff explained the complications preventionmeasures, delirium nursing methods and rehabilitation exercise methods for the patients and their families. Because the patients were in bed for a long time after the operation, it was necessary to prevent the occurrence of stress injury.

### Nutritional support methods

Patients in the CG group received normal dietary intervention. Patients were instructed not to fast or drink water before surgery. After surgery, they were given a small amount of water to drink without discomfort after waking up from anaesthesia. The patients were provided with easily digestible food rich in fibre, high vitamins, and high protein, and they were given less food. Eat more meals. The patient’s serum protein and nutritional score were reviewed after surgery. For patients with low serum protein and a high risk of nutritional score, additional treatment with albumin or oral, enteral nutrition suspension should be followed as directed by the doctor.

Patients in SG received enteral nutritional support from the beginning based on CG. On the firstday after surgery, in addition to the normal diet, enteral nutrition suspension (Nutricia Pharmaceutical (Wuxi) Co., Ltd., National Drug Approval No. H20030012) was taken orally 100 m times, 5 times/day. After 3 days of administration, the dose will be increased to 1000 mL/d according to the patient’s intestinal tolerance. The intervention will last for 7 days. If the patient is intolerant to the intestinal tract, albumin treatment will be added as directed by the doctor on time.

### Observation indicators

(1) The first time out of bed and length of hospital stay after surgery were recorded in 2 groups.

(2) The incidence of postoperative delirium and related adverse events (extubation, pressure injuryand exudation) were recorded and compared between 2 groups. Evaluation criteria for delirium: CAM-ICU was adopted to evaluate the occurrence of postoperative delirium in 2 groups, including 11 items such as attention deficit and confusion of thinking. Each item scored 1–4; the higher the score, the more serious the degree of delirium. The score values of all items were added to the total score, and 20 points could be judged as delirium.

(3) Self-rating anxiety scale (SAS) and self-rating depression scale (SDS) were implemented to evaluate the changes in mental state of 2 groups [14].

(4) The nutritional status of 2 groups was compared. 2 mL of fasting elbow venous blood was extracted from the two groups in the morning, and the levels of haemoglobin (Hb), total protein (TP) as well as albumin (ALB) were determined by an automatic biochemical analyser.

(5) An automatic biochemical analyser detected the changes in serum IgA, IgG, and IgM levels.

(6) The pain degree of 2 groups was compared 1, 3 and 7 days after surgery. VAS scale (0~10 points) was used to evaluate the pain degree and score.

(7) Richards-Campbell Sleep Questionnaire (RCSQ) scale was adopted to assess the sleep quality of 2 groups [15], with a score range of 0–100 points, and the scores were positively correlated with sleep quality.

(8) The self-made nursing satisfaction questionnaire was adopted to assess the nursing satisfaction of the two groups during hospitalisation. 90~100 patients were very satisfied, 80~89 were satisfied,and <79 were dissatisfied. The percentage of the sum of very satisfied cases and satisfied cases to the total number of cases was nursing satisfaction.

### Statistical analysis

SPSS 24.0 statistical software was adopted for data analysis. Measurement data were expressed as(x̄±s), and a t-test was adopted for comparison. Count data were expressed as (n, %), and the χ^2^ test was used for comparison. P<0.05 meant statistical significance.

## Results

### Characteristics of patients included in the study

The CG contained 20 males and 36 females aged 65–88 years, and the average age was(78.64±7.78) years. There were 20 cases of femoral neck fracture, 34 cases of femoral intertrochanteric fracture, and 2 cases of femoral subtrochanteric fracture. The SG contained 21 males and 35 females, aged 66–89 years, and the average age was (78.68±7.82) years. There were 20 cases of femoral neck fracture, 35 of femoral intertrochanteric fracture, along with 1 case of femoral subtrochanteric fracture. No significant difference was discovered in the general data between 2 groups (P>0.05), implying comparable (Table 1).

**Table 1:** Characteristics of patients included in the study.

Variable	Control Group (CG) (n=56)	Study Group (SG) (n=56)	P-value
Age (years)	78.64±7.78	78.68±7.82	0.67
Gender (Male/Female)	20/36	21/35	0.87
*Fracture Type*			
Femoral Neck Fracture	20	20	0.59
Femoral Intertrochanteric Fracture	34	35	
Femoral Subtr ochanteric Fracture	2	1	

### Postoperative recovery and delirium incidence

**Figure 1:**
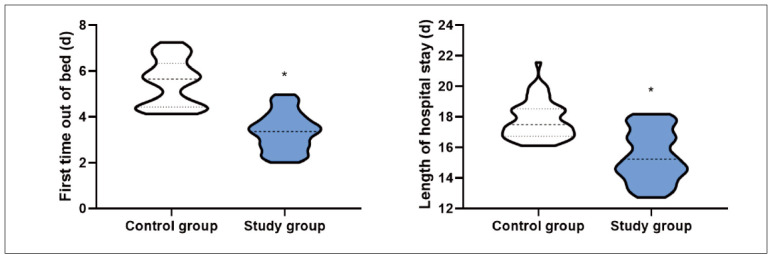
First time out of bed and length of hospital stay after surgery in 2 groups. *P<0.05.

In contrast to the CG, the first time out of bed and length of hospital stay after surgery in the SG presented shorter (P<0.05, Figure 1).

In contrast to the CG, the incidence of delirium and adverse events in the SG presented a reduction (P<0.05, Table 2).

**Table 2:** Incidence of postoperative delirium and related adverse events in 2 groups.

Groups	Cases	Delirium	Adverse events			
			Extubation	Pressure	Exudation	Total incidence rate
Control group	56	8 (14.29%)	3	2	3	8 (14.29%)
Study group	56	1 (1.79%)	0	0	1	1 (1.79%)
χ^2^		5.920				5.920
P		0.015				0.015

### Immune function, nutritional status, and pain management

**Figure 2:**
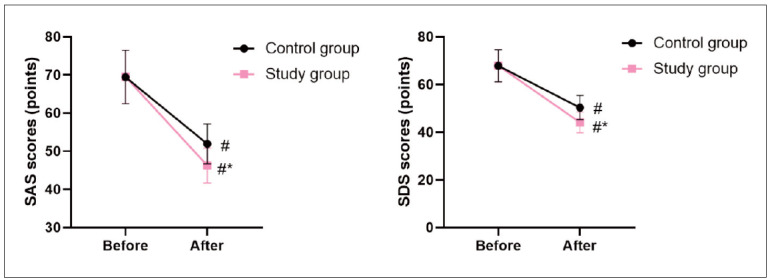
Mental state of patients in 2 groups. In comparison with before intervention, # meant P<0.05. In comparison with CG, * meant P<0.05.

Before the intervention, no difference was seen in SAS and SDS scores between 2 groups (P>0.05). After the intervention, SAS and SDS scores declined in 2 groups, and those in the SG presented lower when compared with the CG (P<0.05, Figure 2).

**Figure 3:**
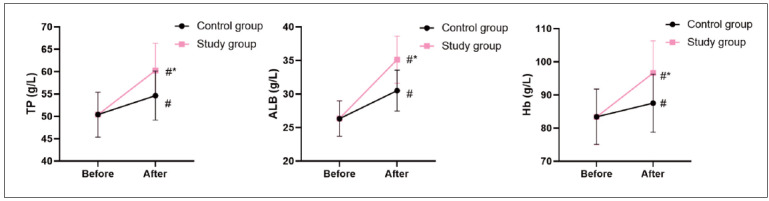
Nutritional status of patients in 2 groups. In comparison with before intervention, # meant P<0.05. In comparison with CG, *meant P<0.05.

Before the intervention, no difference was seen in Hb, ALB and TP levels between 2 groups (P>0.05). After intervention, Hb, ALB and TP levels were elevated in 2 groups, and those in the SG presented higher when compared with the CG (P<0.05, Figure 3).

**Figure 4:**
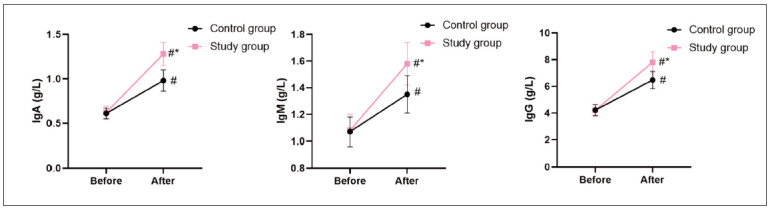
Immune function in 2 groups. In comparison with before intervention, # meant P<0.05. In comparison with CG, *meant P<0.05.

Before the intervention, no difference was seen in IgA, IgM, and IgG levels between 2 groups (P>0.05). After the intervention, IgA, IgM, and IgG levels were elevated in 2 groups, and those in the SG presented higher when compared with the CG (P<0.05, Figure 4).

**Figure 5:**
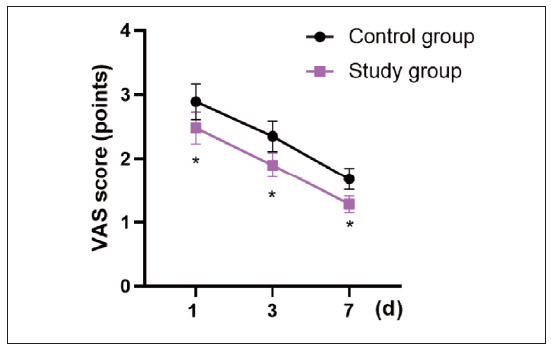
VAS score in 2 groups. *P<0.05.

The VAS score of the SG presented lower than that of the CG on day 1, day 3 and day 7 after surgery (P<0.05, Figure 5).

### Psychological well-being, sleep quality, and patient satisfaction

**Figure 6:**
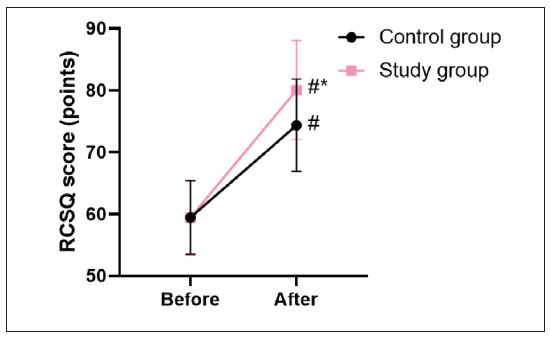
Sleep quality of patients in 2 groups. In comparison with before intervention, # meant P<0.05. In comparison with CG, * meant P<0.05.

Before the intervention, no difference was seen in the RCSQ score between 2 groups (P>0.05). Afterthe intervention, the RCSQ score was elevated in 2 groups, and SG presented higher when compared with the CG (P<0.05, Figure 6).

In contrast to the CG, the nursing satisfaction of patients in the SG presented better (P<0.05, Table 2).

## Discussion

In our study, we observed that nutritional support combined with stratified nursing under a delirium risk assessment strategy significantly enhanced immune function and reduced the incidence of postoperative delirium in elderly hip fracture patients. These findings align with existing literature indicating that malnutrition impairs the immune response, increasing susceptibility to postoperative infections and that poor nutritional status is associated with a higher risk of complications such as pressure ulcers after hip fracture [16]. Furthermore, postoperativedelirium (POD) is a common complication in geriatric patients with hip fractures and is linked to poorer functional recovery and more extended hospital stays [17].

With the gradual ageing of the population in China, the incidence of hip fractures in older people is also increasing [18], and the incidence of postoperative delirium is also growing [19]. When delirium occurs in patients with abnormal behaviour, hyperactivity, and mood swings, it easily leads to extubation, bed falls and other adverse events, which seriously affect the prognosis of patients [20]. Hence, finding an effective nursing measure to prevent related adverse events is particularly crucial.

CAM-ICU is an effective tool for assessing delirium in critically ill patients. According to the score, patients can be classified into high, medium, and low risks, and then targeted nursing intervention can be given [21]. Stratified nursing is based on the classification of patients based on CAM-ICU scoring, and through comprehensive evaluation of patients, risk factors affecting delirium are found, and appropriate nursing interventions are given according to the actual situation of patients, which can effectively improve the bad emotions of patients, decrease the occurrence of delirium and adverse events, along with promote the prognosis of patients [22].

Elderly patients account for a large proportion of hip fractures, and elderly patients are prone to problems such as poor metabolic capacity, slow postoperative recovery, reduced immunity, and poor healing [23]. Based on this, it is essential to offer nutritional support for patients in hip surgery recovery to promote their nutritional status and improve their prognosis.

Enteral nutrition suspension is a compound preparation that contains essential nutrients for the human body and is suitable for enteral nutrition patients who cannot eat normally due to gastrointestinal dysfunction [24]. Besides, the enteral nutrient suspension is a kind of enteral nutrient based on whole protein, which contains comprehensive nutrients and is easy to digest and completely absorbed by the human body, so it has a high bioavailability, which helps to promote the nutritional status and elevate the body’s immunity, and accelerate the recovery of patients [25]. The enteral nutrient suspension contains many amino acid short peptides, whose transport effect helps intestinal mucosa absorb protein, improve the nutritional level of patients, and promote the healing and functional recovery of fracture ends and incisions [26].

In this study, nutritional support combined with stratified nursing based on CAM-ICU scoring strategy was applied to postoperative nursing of patients with osteoporotic hip fracture. It was found that in contrast to the CG, the first time out of bed, as well as length of hospital stay after surgery in the SG, presented shorter, the incidence of delirium and adverse events in the SG presented reduction, and the VAS score of the SG presented lower, suggesting that nutritional support combined with stratified nursing under delirium risk assessment strategy could promote the recovery and reduce the occurrence of delirium in elderly hip fracture patients after surgery. Similar studies have highlighted the importance of nutritional status in postoperative outcomes. For instance, research indicates that malnutrition is a significant risk factor for postoperative delirium (POD) in elderly hip fracture patients. The Prognostic Nutritional Index (PNI), a tool used to assess nutritional status, has been validated in this context, underscoring the critical role of nutrition in patient recovery [27]. Furthermore, evidence supports that nutritional support can reduce the length of hospital stay, morbidity, mortality, and the incidence of delirium. The Geriatric Nutritional Risk Index (GNRI) has been identified as a predictor of POD, emphasising the need for nutritional interventions in this patient population [28].

Besides, our study indicated that SAS and SDS scores declined in 2 groups after the intervention. Those in the SG presented lower when compared with the CG, suggesting that nutritional support combined with stratified nursing under a delirium risk assessment strategy could relieve the negative emotions of elderly hip fracture patients after surgery, which was following the previous study [29].

The results of our study indicated that after intervention, Hb, ALB and TP levels in the SG presented higher when compared with the CG, IgA, IgM and IgG levels in the SG presented higher when compared with the CG, and RCSQ scores in the SG presented higher when comparing with the CG, implying that nutritional support combined with stratified nursing under delirium risk assessment strategy could promote the nutritional status, immune function and sleep quality of elderly hip fracture patients after surgery. Many studies have consistently proved that enteral nutrition can promote the nutritional status, immune function, and sleep quality of patients undergoing surgery [30] [31].

In addition, our study indicated that in contrast to the CG, the nursing satisfaction of patients in the SG presented better, indicating a high degree of recognition of the application of nutritional support combined with stratified nursing under a delirium risk assessment strategy.

## Conclusion

This study confirms that nutritional support combined with stratified nursing under delirium risk assessment enhances immune function and reduces postoperative delirium in elderly hip fracture patients. The intervention also improves nutritional status, shortens hospital stays, and accelerates recovery, leading to better postoperative outcomes. These findings suggest that integrating targeted nursing and nutritional strategies can effectively optimise patient care and recovery.

## Dodatak

### Conflict of interest statement

All the authors declare that they have no conflict of interest in this work.
